# Fracture resistance of CAD/CAM-milled, 3D-printed, and customized fiber post-and-core systems: an in vitro study

**DOI:** 10.1186/s12903-025-07418-9

**Published:** 2025-12-11

**Authors:** Hoda Alaa El-Din Selim El-Shorbagy, Ghada A.E. Hussien, Hoda M. Abel Sadek, Amina M. Hamdy

**Affiliations:** 1 Fixed prosthodontics department, Faculty of Dentistry, Ain Shams University, El-Khalyfa El-Mamoun Street Abbasya, Cairo, Egypt; 2 Fixed Prosthodontics department, Faculty of Dentistry, Galala University, Suez, Egypt

**Keywords:** Custom-made post, CAD/CAM, Customized fiber post, Milled post, Fracture resistance, Failure mode

## Abstract

**Background:**

Post-and-core systems are frequently used when restoring endodontically treated teeth. CAD/CAM technologies contribute to post fabrication by improving material uniformity and adaptation to canal morphology. Additionally, 3D printing presents an emerging approach that constructs posts incrementally and may address certain limitations associated with traditional fabrication methods. The purpose of this in vitro study was to evaluate the fracture resistance of custom-made CAD/CAM posts and cores fabricated using milling and additive techniques compared to customized fiber posts.

**Materials and methods:**

Twenty-one maxillary central incisors were randomly divided into three groups (*n* = 7): Group MP (milled posts), Group 3D (3D printed posts), and Group CF (customized fiber posts). The teeth were embedded in acrylic resin and prepared endodontically. Post spaces were created using standardized drills. For Groups MP and 3D, the post spaces were scanned, and posts were fabricated using CAD/CAM technology and 3D printing. In Group CF, posts were customized with resin composite. All posts were cemented with resin cement. The specimens were subjected to thermocycling for 5,000 cycles. Fracture resistance was measured using a universal testing machine, and failure modes were examined with a stereomicroscope. The Kruskal-Wallis test was used to compare the three groups. Failure mode data were reported as frequencies and percentages. Fisher’s Exact test was used to compare failure modes among the three groups.

**Results:**

There was no statistically significant difference in fracture resistance among the three groups (*p* = 0.400) or in failure modes (*p* = 0.381). The 3D group exhibited the highest percentage of restorable failures (85.7%), while the Milled group showed the highest percentage of non-restorable failures (57.1%).

**Conclusions:**

All post types demonstrated comparable fracture resistance and favorable failure modes when restoring anterior endodontically treated teeth. Milled composite blocks and 3D-printable materials effectively produced one-piece post-and-core structures using CAD/CAM technology with fracture resistance falling within the typical masticatory forces for maxillary anterior teeth.

## Background

Teeth that have undergone endodontic treatment are more prone to fracture than vital teeth due to the substantial loss of tooth structure [1]. The primary factors contributing to this loss include dental caries, defective restorations, endodontic access preparation, and root canal preparation [2, 3]. When restoring devitalized teeth, dental materials must be capable of compensating for this structural loss to ensure the restoration has appropriate mechanical and functional properties, esthetics, and a preserved coronal seal [4]. A post and core may be required in endodontically treated teeth with extensive loss of coronal structure, as the post functions primarily to retain the core material, which in turn provides the necessary retention and resistance form for the final full coverage restoration, rather than reinforcing the remaining tooth structure [5]. The post and core material should possess mechanical and physical properties similar to dentin to effectively withstand occlusal forces, reducing the risk of tooth fracture or post debonding [6, 7]. The post and core should exhibit a modulus of elasticity, compressive strength, and coefficient of thermal expansion like that of dentin in order prevent further destruction to already compromised teeth [8].

According to the method of construction, there are two types of posts that can rehabilitate a root canal and provide retention for a crown: custom-made posts and prefabricated posts [9]. A custom-made post is created by taking an impression directly from the root cavity [10]. The custom-made post and core offer a precise fit to the canal wall and effectively resist torsional forces [11]. It is especially indicated in cases with insufficient ferrule or an irregularly shaped canal [12]. Prefabricated post is commercially available in various geometries, dimensions, and materials. The advantages of prefabricated posts include the ability to complete the restoration in a single visit, resulting in quicker, less expensive treatment with good retention to the tooth [13].

Anatomical post technique is recommended for the rehabilitation of anterior teeth with a significantly destroyed canal and a considerable loss of hard tissue [14]. The anatomical post offers a new alternative to custom posts in these cases, where an esthetic fiberglass post is placed, externally surrounded by direct composite resin, ensuring a perfect adaptation [15].

A custom post and core refers to a post-and-core system that is made for an individual root canal [11]. A custom-made post, typically fabricated from cast alloy, can lead to stress concentrations within the surrounding radicular dentin, which may result in root fractures and non-restorable failure [16]. The use of computer aided design and computer-aided manufacturing (CAD/CAM) makes it possible to produce a single-piece post and core, reducing the number of interfaces between the resin composite core and the fiberglass post [17]. This, in turn, decreases the likelihood of structural failure of the material [6, 18]. CAD/CAM technology has revolutionized the fabrication of posts and cores, enhancing reliability and marginal adaptation compared to conventional methods [19]. New CAD/CAM materials have been developed by combining ceramics with composite resins [15]. Examples of such hybrid materials include VITA Enamic, consisting of 75% feldspathic porcelain and 25% composite resin, along with Lava Ultimate (3 M, St. Paul, MN, USA), which consists of composite resin embedded with 80% nano-ceramic particles [20]. Newer materials, such as Polyetheretherketone (PEEK), have also been reported for the fabrication of CAD/CAM posts and cores, as their relatively low modulus of elasticity helps reduce stress concentration [6, 21].

Three-dimensional (3D) printing, also known as additive manufacturing, is gaining popularity in dentistry due to advancements in materials and printer technology [22]. It helps overcome the limitations of subtractive manufacturing methods, such as geometric restrictions that prevent fabrication of complex internal features, excessive material waste during milling, tool wear that affects precision, limited compatibility with flexible materials, and low productivity in batch production [23]. It is frequently used in the production of dental implants, orthodontic models, metal restorations, implant surgery guides, and temporary crowns [24]. With the ongoing advancement of light-curing resin composite materials, adhesively cemented permanent single-tooth restorations can now be printed [25]. A key requirement for printable materials is maintaining a flowable consistency while ensuring structural stability during both printing and storage [26]. To ensure stable liquid consistency, printable dental materials for permanent restorations should contain fewer inorganic fillers than those used in blocks and discs, similar to flowable dental composites [26]. This lower filler content impacts the material’s stiffness, resulting in an E-modulus value significantly lower than that of hard tissues [27]. VarseoSmile Crown Plus (VS) is a newly developed, tooth-colored, ceramic-filled hybrid resin material that is light-cured and based on methacrylic acid esters [28]. These materials can withstand high occlusal forces, making them suitable for the manufacture of inlays, onlays, veneers, and permanent crowns [28].

Based on the literature review, there is limited evidence regarding the efficacy of using three-dimensional (3D) printing techniques for the construction of custom-made posts compared to milled posts. Therefore, the objective of this study was to evaluate the fracture resistance of endodontically treated teeth restored with milled and 3D printed custom-made posts and cores, compared to contemporary customized fiber posts. The null hypothesis is that there is no difference in the fracture resistance of endodontically treated anterior teeth restored with custom-made posts and cores fabricated using milling or 3D printing techniques, or with customized fiber posts.

## Materials

Information on the materials, compositions, manufacturers, and lot numbers used in the study are listed in Table 1.

**Table 1:** Information on the materials, compositions, manufacturers, and lot numbers used in the study

Trade name	Composition	Manufacturer	Lot no.
Varseo Smile Crown plus (VS)	- Silanized dental glass, methyl benzoyl formate, diphenyl (2,4,6-trimethyl benzoyl) phosphine oxide. - 40-isopropy lidiphenol, ethoxylated and 2-methylprop-2enoic acid. -Filler mass (0.7 μm particle size forming 30–50 wt% inorganic filler)	Bego, Bremen, Germany	600,968
Brilliant Crios (BC) blocks size 14 (A3 LT)	- 70.7% <20 nm Amorphous silica and < 1 μm barium glass. - 29.3% Cross-linked methacrylate resin matrix (Bis-GMA, Bis-EMA, TEGDMA) -Filler mass ((SiO2 < 20 nm, barium glass < 1 μm forming 70.7 wt% inorganic filler)	Coltène, Whaledent A.G, Altstatten, Switzerland.	J46379
Itena dentoclic fiber post	- Glass fiber post - 80% unidirectional parallel oriented glass fibers embedded in 20% of epoxy-resin	ITENA, France	54,674/1
Amaris Composite resin	- Inorganic fillers (80% w/w) - Methacrylate matrix (Bis-GMA, UDMA, TEGDMA)(20% w/w)	VOCO, Germany	1,424,079–1,426,517
LuxaCore Z	-Core buildup material -Bis-GMA -Nanofiller and Zirconium dioxide -Barium glass -Pyrogenic silicic acid	DMG, Germany	243,872
G-CEM capsules	-Dual-cure resin cement -UDMA3-7% -Dimethacrylate30-60% w/w -Phosphoric acid ester monomer1-5% w/w -Stabilizer ≤ 1% w/w -Hydroquinone (HQ) ≤ 1% w/w -fluoro alumino silicate glass -Photoinitiator ≤ 1% w/w	GC corporation, Japan	1,908,031

## Methods

### Sample size calculation

To determine the number of specimens that would be required for each test group, power analysis was conducted. Power analysis was designed to have adequate power to apply a statistical test of the null hypothesis that there was no difference between different tested groups regarding fracture resistance. By adopting an alpha (α) level of 0.05 (5%), a beta (β) level of 0.20 (20%) (i.e. power = 80%), and using an effect size (f = 0.738) calculated based on the results of a previous study [29]; the minimum required sample size (n) was found to be (21) samples (i.e. 7 samples per group). Sample size calculation was performed using G*Power version 3.1.9.2 [30].

**Fig. 1:**
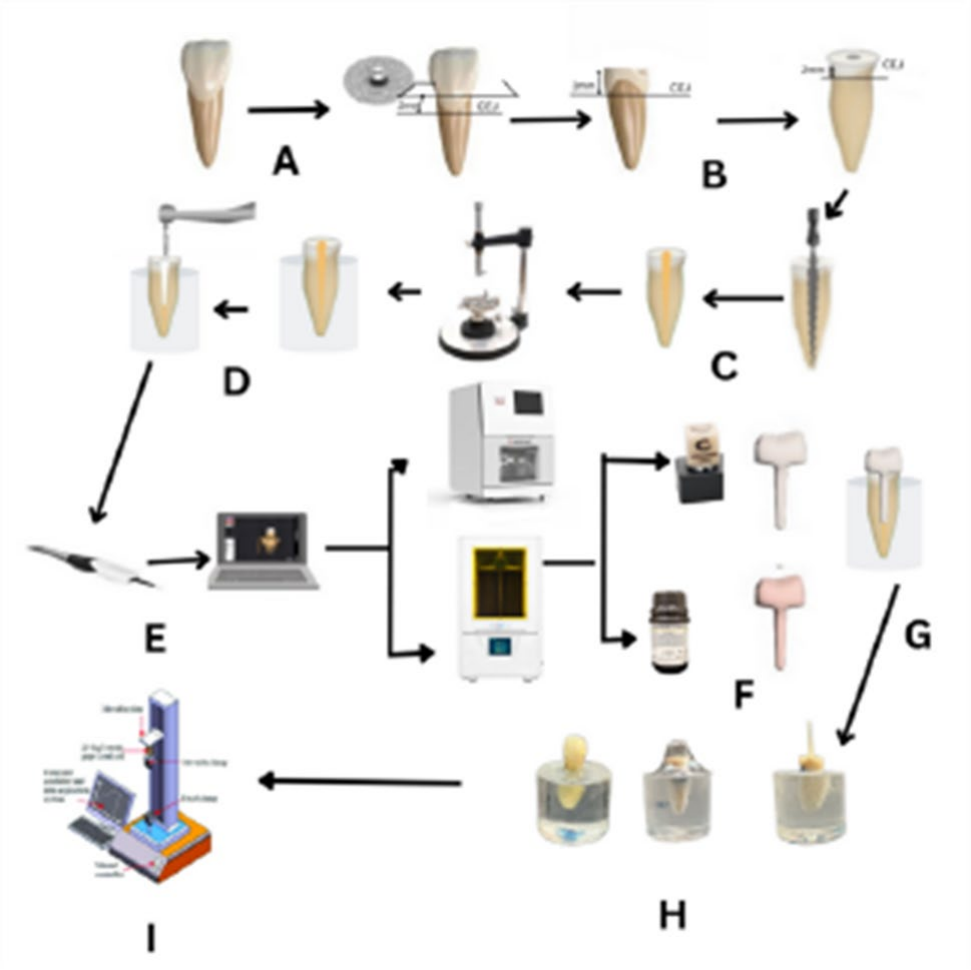
Schematic diagram showing the workflow of the methodology; **A**: extracted maxillary central incisor, **B**: tooth decoronation, **C**: root canal treatment, **D**: mounting in epoxy resin block and drilling post space, **E**: scanning of the post space, **F**: construction of milled and 3 d printed post and core, **G**: post and core cementation, **H**: customized fiber post construction using silicone template, **I**: fracture resistance testing

A complete summarized workflow of methodology is shown in schematic diagram, Fig. 1.

### Selection of teeth, teeth decoronation and endodontic treatment

This study was approved by the Ethics in Research Committee (FDASU-RecEM122241 approval number). A total of 21 human maxillary central incisors with similar shape and root form were selected. The teeth were collected from the Diabetes Institute, and their owners could not be identified, extracted for periodontal reasons and were included if they met the following criteria: fully developed apices, no caries, cracks, restorations, erosion, abrasion, or fractures. The coronal size at the cervical line ranged from 6 ± 1 mm bucco-lingually and 5 ± 1 mm mesiodistally. The crown length was approximately 10 ± 0.5 mm, and the root length was 12.5 ± 0.5 mm. All dimensions were measured using a digital caliper (Digital Caliper, CEN-TECH, Virginia, USA). Periapical radiographs were taken to confirm the straight pathway of the root canals. Teeth that did not meet these specifications were excluded. The teeth were carefully cleaned, and all external debris was removed using an ultrasonic scaler. Then, the extracted human teeth were stored in distilled water at room temperature and used within a period of less than six months from extraction, in accordance with ISO 11405:2015 guidelines, to preserve dentin integrity and simulate intraoral conditions as recommended in previous study [31]. Subsequently, the crowns were sectioned 2 mm above the cemento-enamel junction with a diamond disc (Komet Dental, Gebr. Brasseler GmbH & Co., Germany) mounted on a straight handpiece under continuous irrigation, to standardize a uniform 14-mm measurement from the apex to the sectioned surface above the CEJ. The collected teeth were endodontically prepared by a single operator using a rotary-files (M-Pro IMD, Guangdong, China) to estimated working length with copious irrigation 5% sodium hypochlorite after each file. For standardization, gutta-percha point was placed to working length with epoxy resin sealer (Adseal Plus). A 40/6% taper fine tip plugger (Fast Pack Pro, Eighteeth) was heated to 200 °C and taken to a depth 3-mm short of working length. The tip was allowed to cool for 15 s, and single burst of heat was applied for one second and the tip was removed. The canal was completely backfilled with (Fast Fill, Eightieth) in many continuous movements to avoid voids formation until the canal was completely obturated with gutta-percha. Each root was then embedded in acrylic resin (Acrostone, cold cure resin, Egypt) up to 2.0 mm short of the cemento-enamel junction, within a circular polyvinyl chloride cylinder (25 mm diameter, 20 mm height) using dental surveyor (Dental Fix, Canada). The set (tooth, matrix, and resin) was allowed to stabilize for 72 h to ensure complete resin setting.

### Post space preparation

The post space preparation was performed using size #2 and #3 Pesso reamers (MANI, Japan) to remove 9 mm of gutta-percha from each tooth. The drilling was done parallel to the long axis of the tooth. To standardize the post space, the preparation continued with fiber post drills (ITENA Dentoclic, ITENA, France) creating a post space with a diameter of 1.3 mm at the apical end using the yellow drill. All procedures were conducted by the same operator (principal investigator) to ensure standardization.

### Teeth grouping and sample construction

The twenty-one teeth were randomly allocated into three groups to receive different types of posts using simple randomization. The groups were as follows: Group MP (Milled posts, *n* = 7), Group 3D (3D printed posts, *n* = 7), and Group CF (Customized fiber posts, *n* = 7).

**Fig. 2:**
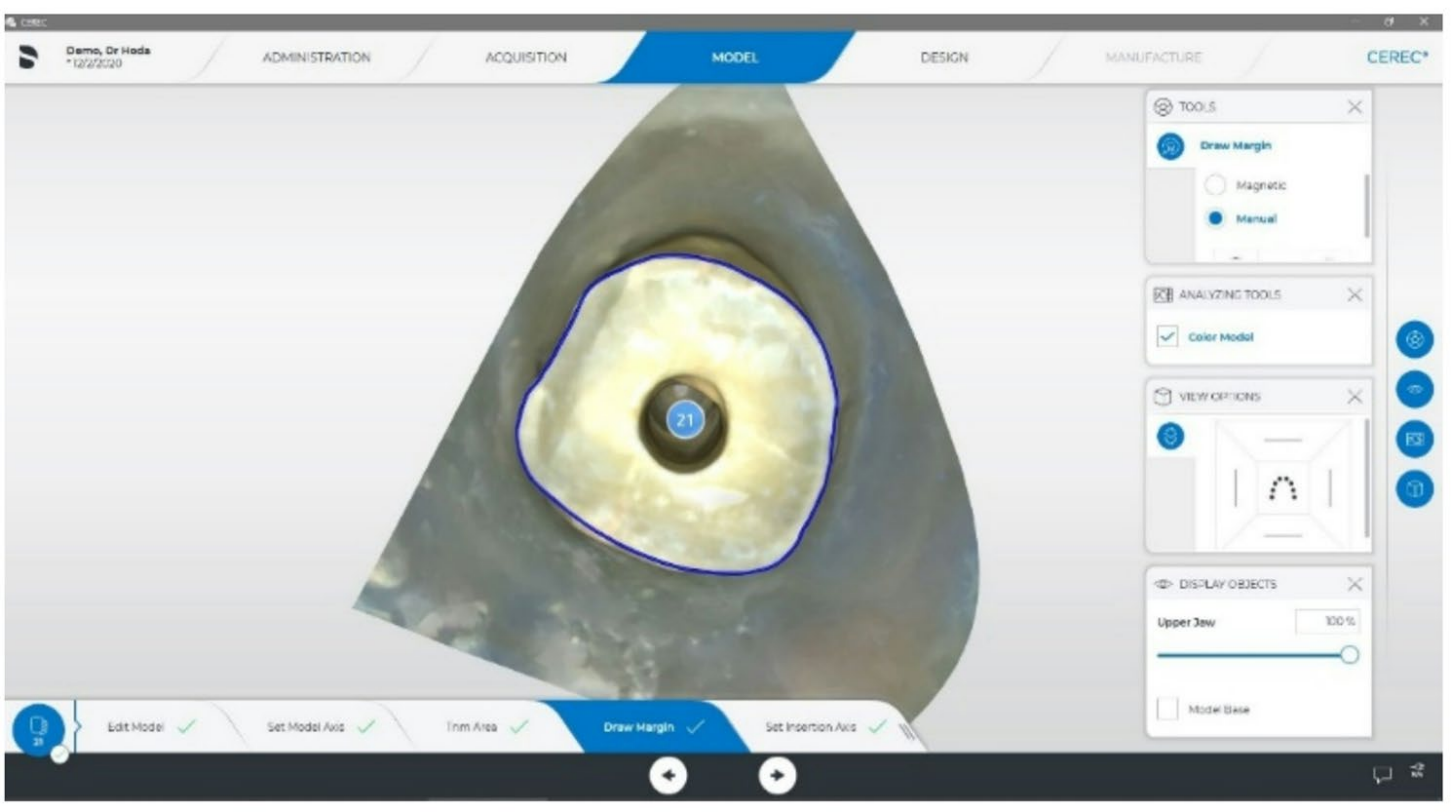
Intracanal and cervical margin determination

**Fig. 3:**
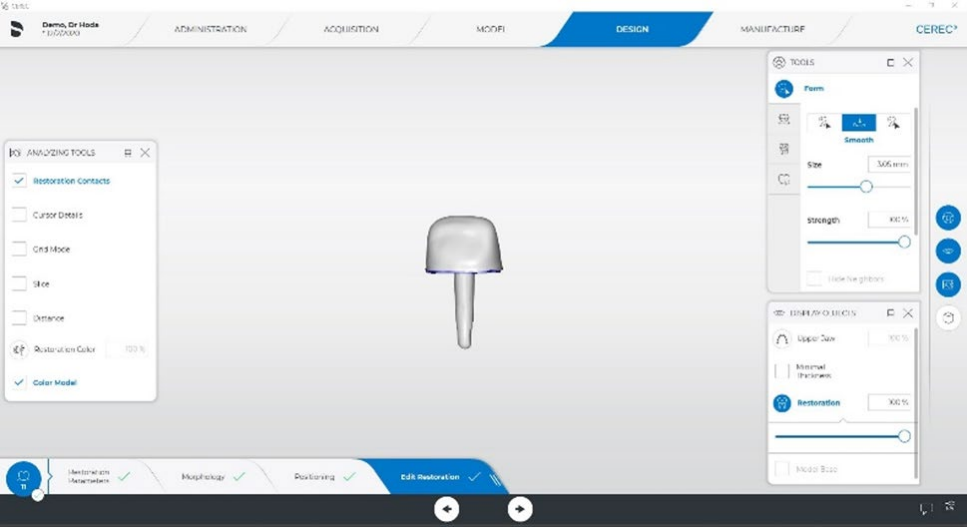
Restoration parameters

The post space of the 14 samples assigned to Groups MP and 3D was scanned using an intraoral scanner (Primescan Intraoral Scanner, Dentsply Sirona, Bensheim, Germany; CEREC 4.5 confocal microscopy). A virtual model of the 3D post and core was created, and a custom-made post and core was designed using CAD/CAM technology with the CEREC system. Key parameters such as margins, core height, post length, and cement space were defined. The core margin was designed to adapt to the cervical margin of the sample (Fig. 2), the core height was set to 5 mm (Fig. 3), and the cement space was 40 μm [32].

For the milled groups (MP), the finished STL file was exported to a 5-axis CAD-CAM milling machine (McCall milling machine D52) to fabricate the custom-made post and cores using Brilliant Crios blocks (size 14, A3 LT, 14 × 12 × 18 mm).

For the 3D printed group (*n* = 7), Using the 3D printer software “PreForm” a layer thickness of 0.05 mm was chosen to achieve the highest level of accuracy with optimal speed. The Model Builder module of the dental CAD software (3Shape, Copenhagen, Denmark) was used to adjust the STL files of the scanned teeth before printing. The finalized STL files were printed using DLP technology on a 3D printer (Anycubic Photon S, Shenzhen, China) with the liquid material VarseoSmile Crown Plus (VS). The 3D printing software Chitubox V1.9.0 (Shenzhen, China) was used to prepare the resin, with optimized settings including a layer height of 0.05 mm for maximum accuracy and speed. Additional settings included 8 bottom layers, 6.5 s exposure time, 20 s bottom exposure, a 5 mm lift distance, and a 60 mm/sec lift speed. The samples were printed vertically on the platform. After printing, the 3D printed samples were carefully removed from the build platform and cleaned according to the manufacturer’s recommendations using ethanol (96%) in an unheated ultrasonic bath (Anycubic 3D Printer Wash and Cure Machine 2.0). The cleaning process was performed for 3 min in the ethanol solution. Afterward, the samples were removed from the ethanol bath and sprayed with additional ethanol (96%) to ensure the complete removal of any residual resin. The samples were then air-dried using compressed air.

Post-curing of the printed samples was conducted twice for 45 min each, using an ultraviolet light curing device (Anycubic 3D Printer Wash and Cure Machine 2.0), which is specifically designed for post-curing 3D printed composite resin materials.

**Fig. 4:**
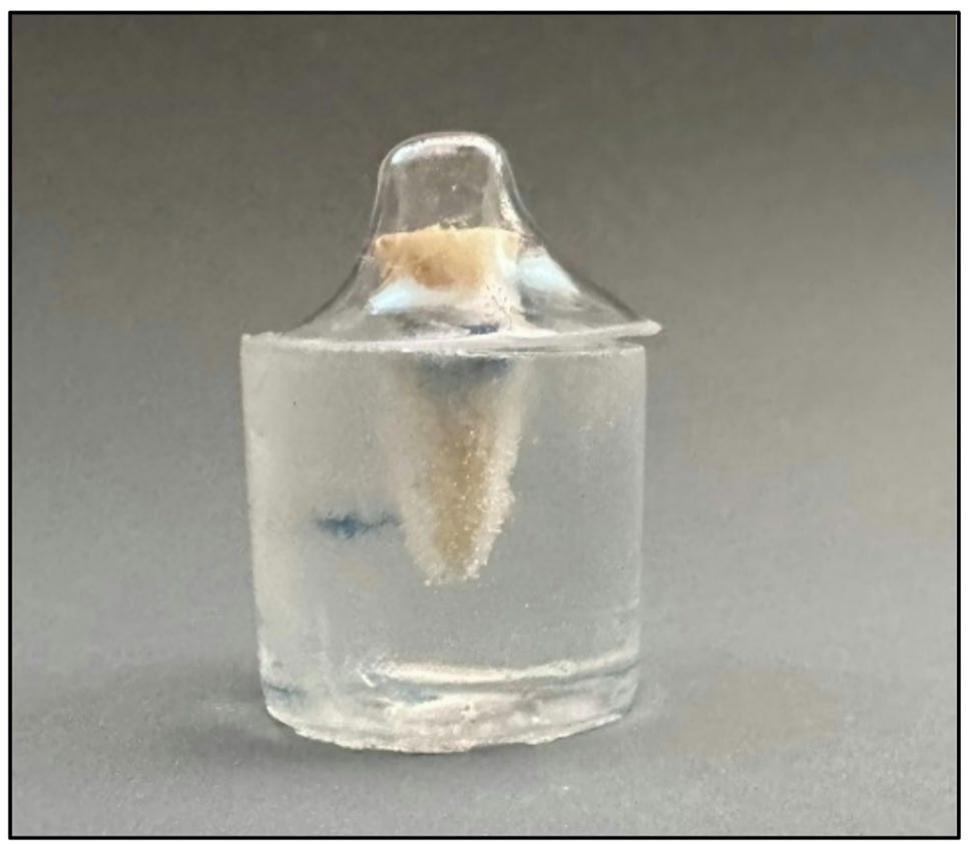
Silicone template fitting

For the Customized Fiber Post group (Group CF), the post surfaces were cleaned by immersion in 70% alcohol for one minute and then dried using sterile gauze. A layer of silane (Dentsply Maillefer, Petrópolis, RJ, Brazil) was applied to the surface of the post for one minute and then allowed to dry before applying the adhesive. Customization was performed by directly applying a micro-hybrid resin composite (Amaris, VOCO, Germany) to the post surface. The post/composite assembly was then inserted into the root canal, which had been previously coated with a water-soluble gel (KY Gel, Johnson & Johnson, São José dos Campos, SP, Brazil) using a micro-brush applicator. Light activation was performed for 5 s, after which the assembly was lifted from the root canal and light-activated for an additional 40 s. After the customization process was complete, the fiber post head was trimmed 4 mm above the coronal end of the specimen to allow for core fabrication. To standardize the size and shape of the composite core, a mold was created using transparent silicone material (polycarbonate pattern, Nucleojet, Ângelus). The mold was made over one of the milled cores from the previous group (Group MP). The template was fabricated in a vacuum forming machine on the Brilliant Crios core and then checked on the teeth of this group (Group CF), to ensure proper fit (Fig. 4). Core build-up material (LuxaCore Z, DMG, Germany) was injected into the template, which was then placed on the customized fiber post. Curing was performed for 40 s light application to all surfaces according to the manufacturer’s instructions.

### Surface treatment of posts

For MP and 3D groups, Air abrasion was performed, with the posts being blasted with 110 μm Al₂O₃ at a pressure of 1.5 bar. The blasting distance was set to 3 cm, in accordance with the manufacturer’s instructions. The restorations were then thoroughly cleaned in an ultrasonic cleaner (CD-4820, CODYSON, Guangdong, China).

### Cementation of posts

Prior to cementation, the root canals were flushed with 2mL of distilled water to remove the residues of water-soluble gel [29]. The canals were dried using triple syringe with oil free air, to ensure complete drying absorbent paper cones (DentsplyMaillefer) were used. A self-adhesive dual-cure resin cement (GCEM Capsules, GC Corporation, Japan) was injected into the root canals to bond the posts to the corresponding tooth surfaces in all tested groups, using a narrow tip to ensure precise application and minimize voids. The restorations were seated with light finger pressure, and excess cement was removed after 5 s of initial polymerization. Full curing was then performed for 40 s at the cervical part of the tooth and on all surfaces using a light cure (3 M ESPE Elipar Deep Cure L LED Polymerization Lamp, Germany) with an energy dose of 800 mW/cm².

### Thermocycling

All specimens were subjected to thermocycling with temperatures of 5 °C and 55 °C (15 s cold, 15 s warm, and 5 s water drip) for 5000 cycles in an automated thermocycling machine (Julabo GmbH, Germany), simulating approximately 6 months of clinical service. After completing the cycling, the specimens were stored in distilled water at room temperature until the fracture test was conducted.

### Fracture resistance testing

**Fig. 5:**
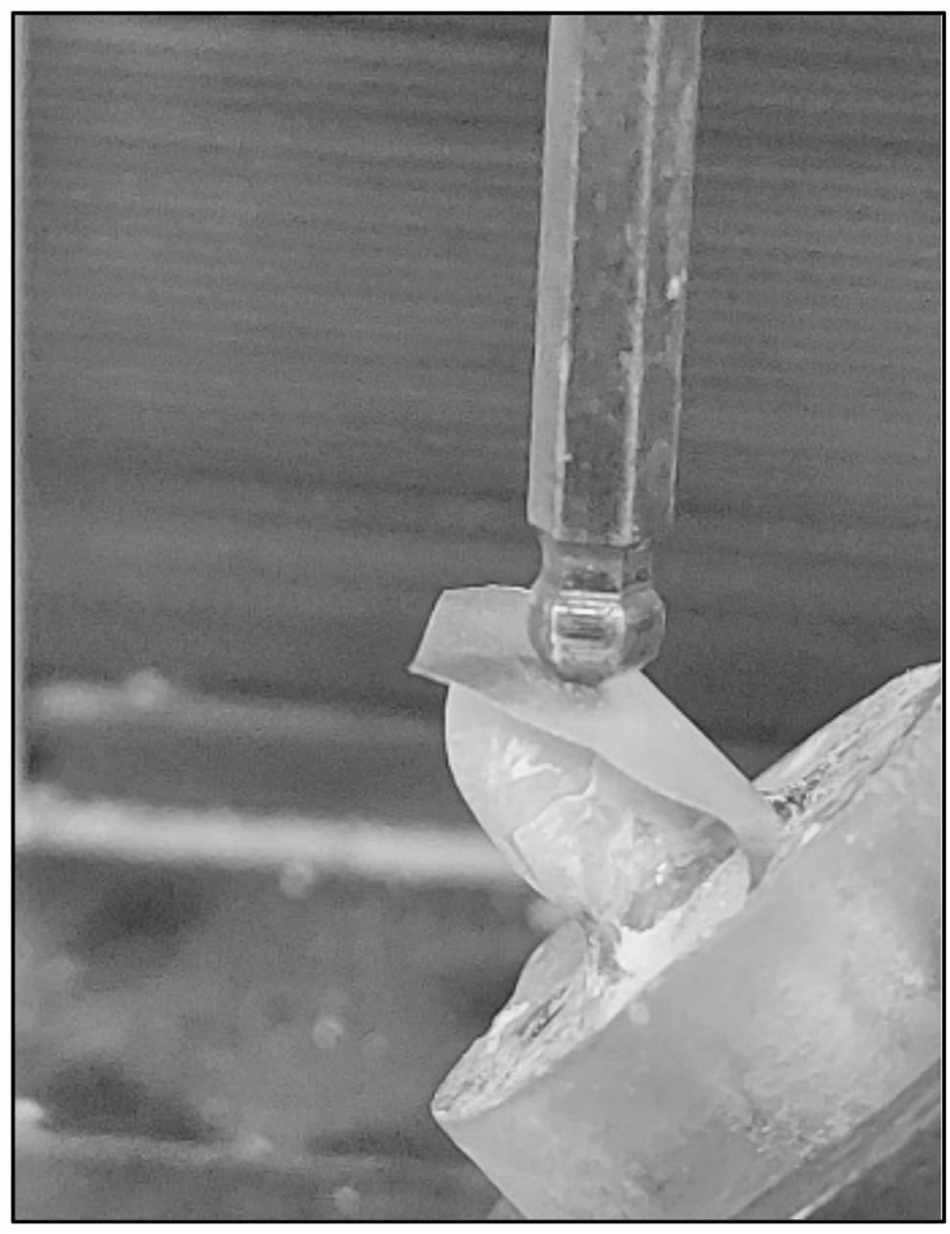
Fracture resistance testing

Each specimen was attached to the lower fixed head at a 45-degree angle to the long axis of the upper loading head of the universal testing machine (Instron Model 3345, England) (Fig. 5), positioned 3 mm from the incisal edge. A thin foil was placed between the load and the teeth to ensure even distribution of force. A compressive force was applied at a crosshead speed of 1.0 mm/min using 5 mm in diameter stainless steel indenter until specimen failure occurred. Failure was indicated by an audible crack and confirmed by a sharp drop on the load-deflection curve, which was recorded using computer software. The force required for failure (in Newtons) was recorded by the machine software (BlueHill Universal, Instron, England).

### Mode of failure

To determine the mode of failure, the failed samples were examined using stereomicroscopic analysis at 20x magnification (SZ1145TR, Olympus, Japan 1990) using software (ToupView, version 3.7). Fracture types were categorized as restorable if the fracture line was above the cemento-enamel junction (simulated bone level) or as unrestorable if the fracture occurred below the cemento-enamel junction (simulated bone level) [33, 34].

### Statistical analys

Quantitative data were assessed for normality by analyzing the data distribution and using normality tests (Kolmogorov-Smirnov and Shapiro-Wilk tests). The fracture resistance data showed a non-normal (non-parametric) distribution. The data were presented as median, range, mean, and standard deviation (SD) values. The Kruskal-Wallis test was used to compare the three groups. Mode of failure data (categorical data) were presented as frequencies and percentages. Fisher’s Exact test was used to compare failure modes between the three groups. A one-sample proportion test was used to compare the modes of failure within each group. The significance level was set at *P* ≤ 0.05. Statistical analysis was performed using IBM SPSS Statistics for Windows, Version 23.0 (Armonk, NY: IBM Corp.).

## Results

### Fracture resistance (N)

**Fig. 6:**
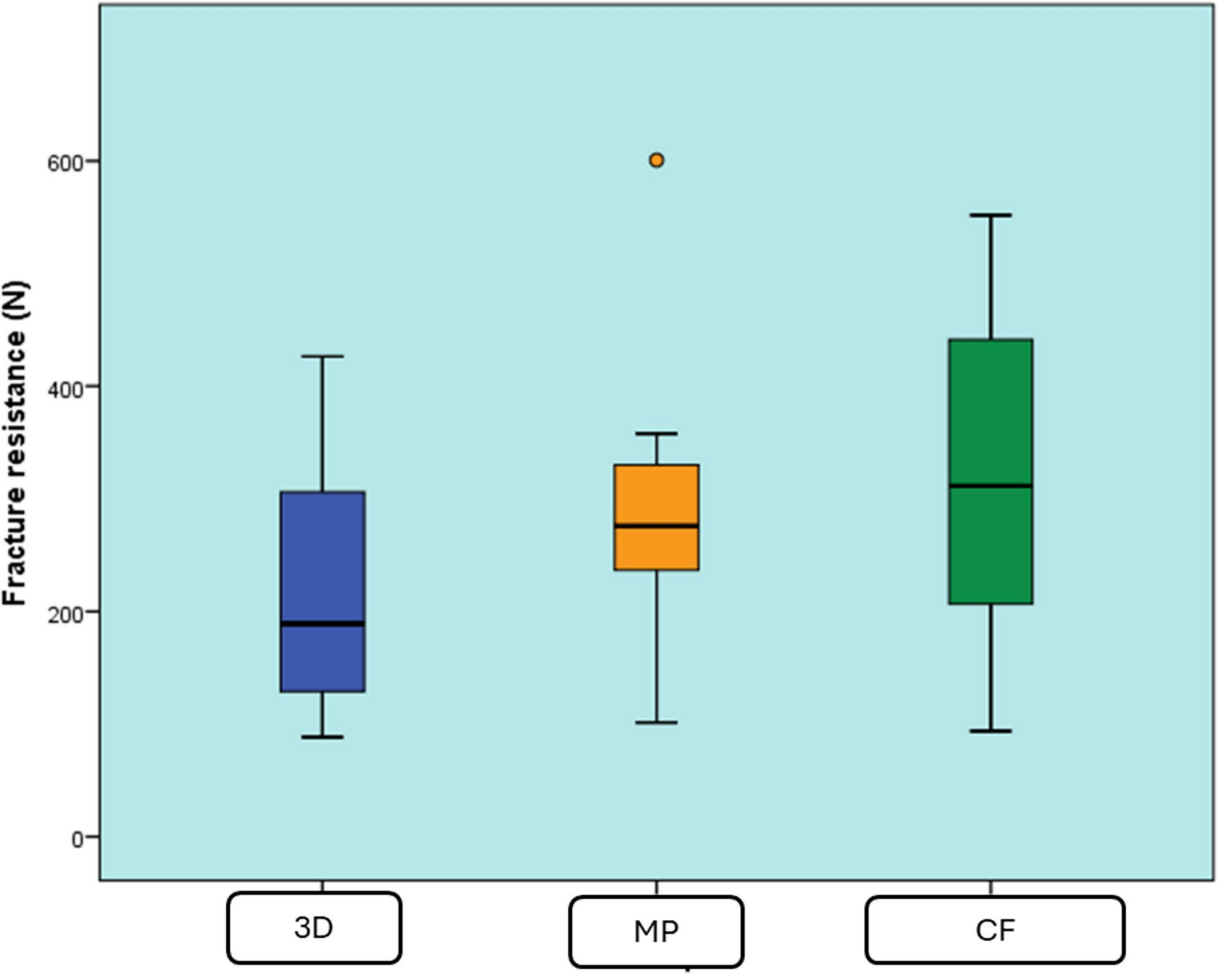
Box plot representing median and range values for fracture resistance of the three groups (Circle represents outlier)

Data were presented as median, range, mean, and standard deviation (SD) values. The Kruskal-Wallis test was used to compare the three groups. The significance level was set at *P* ≤ 0.05 (Table 2; Fig. 6). The results showed no statistically significant difference between the fracture resistance values of the three groups (P-value = 0.400, effect size = 0.079).

**Table 2:** Descriptive statistics and results of Kruskal-Wallis test for comparison between fracture resistance (N) of the three groups

Group	Median	Range	Mean	SD	*P*-value	Effect size (Eta squared)
3D Printing (3D)	189.2	88.5–426.3.5.3	224.8	140.9	0.400	0.079
CAD/CAM (MP)	276	101.3–600.7.3.7	301.6	154.6		
Customized fiber post (CF)	311.5	93.7–551.7.7.7	321.9	165.3		

*: Significant at P ≤ 0.05

### Mode of failure

#### Comparison between three groups

**Fig. 7:**
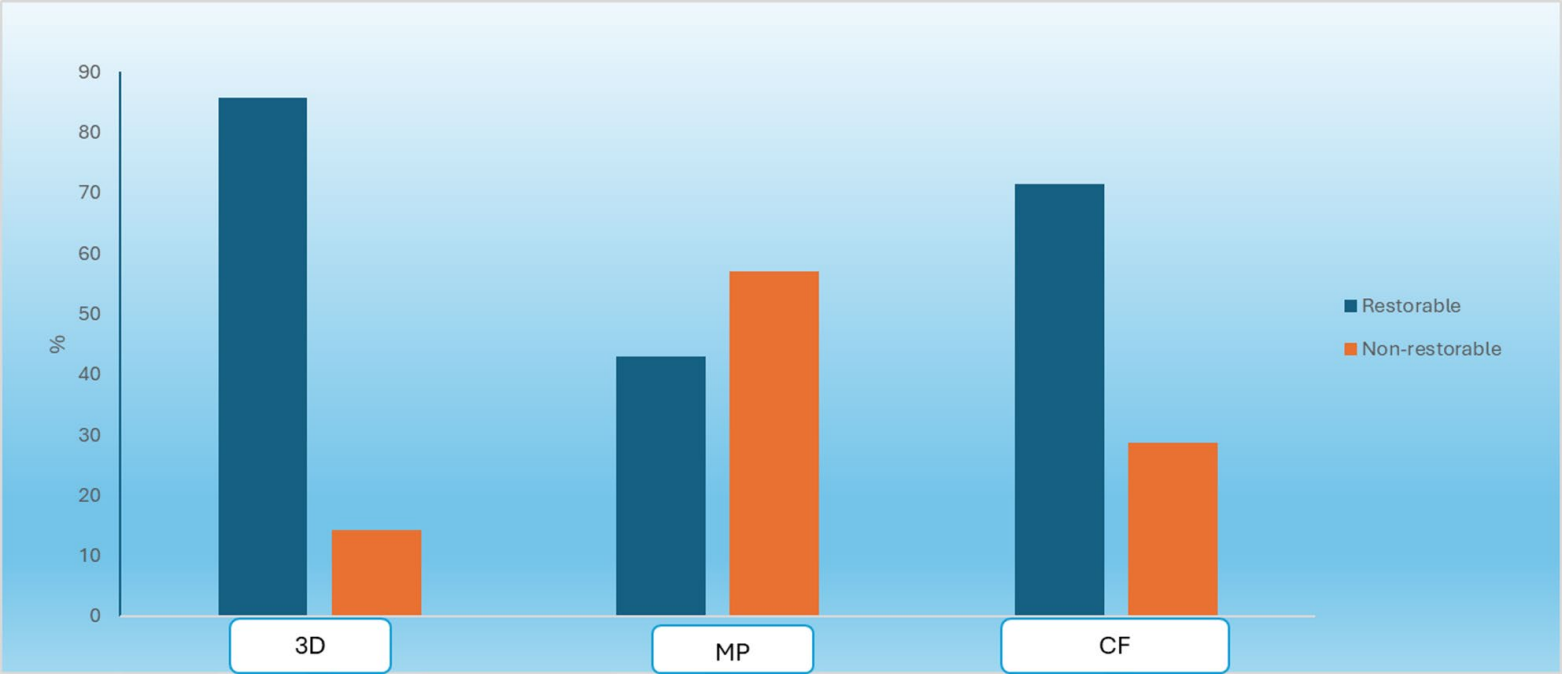
Bar chart representing distribution of failure modes in the three groups

Mode of failure data (categorical data) were presented as frequencies and percentages. Fisher’s Exact test was used to compare the failure modes between the three groups (Table 3; Fig. 7). The significance level was set at *P* ≤ 0.05.

**Table 3:** Frequencies (n), percentages (%) and results of fisher’s exact test for comparison between failure modes of the three groups and one sample proportion test for comparisons within each group

Failure mode	3D Printing		CAD/CAM		Customized fiber post		*P*-value	Effect size (v)	
	*n*	%	*n*	%	*N*	%			
Restorable	6	85.7	3	42.9	5	71.4	0.381		0.378
Non-restorable	1	14.3	4	57.1	2	28.6			
*P*-value	0.125		1		0.453				

*: Significant at P ≤ 0.05

**Fig. 8:**
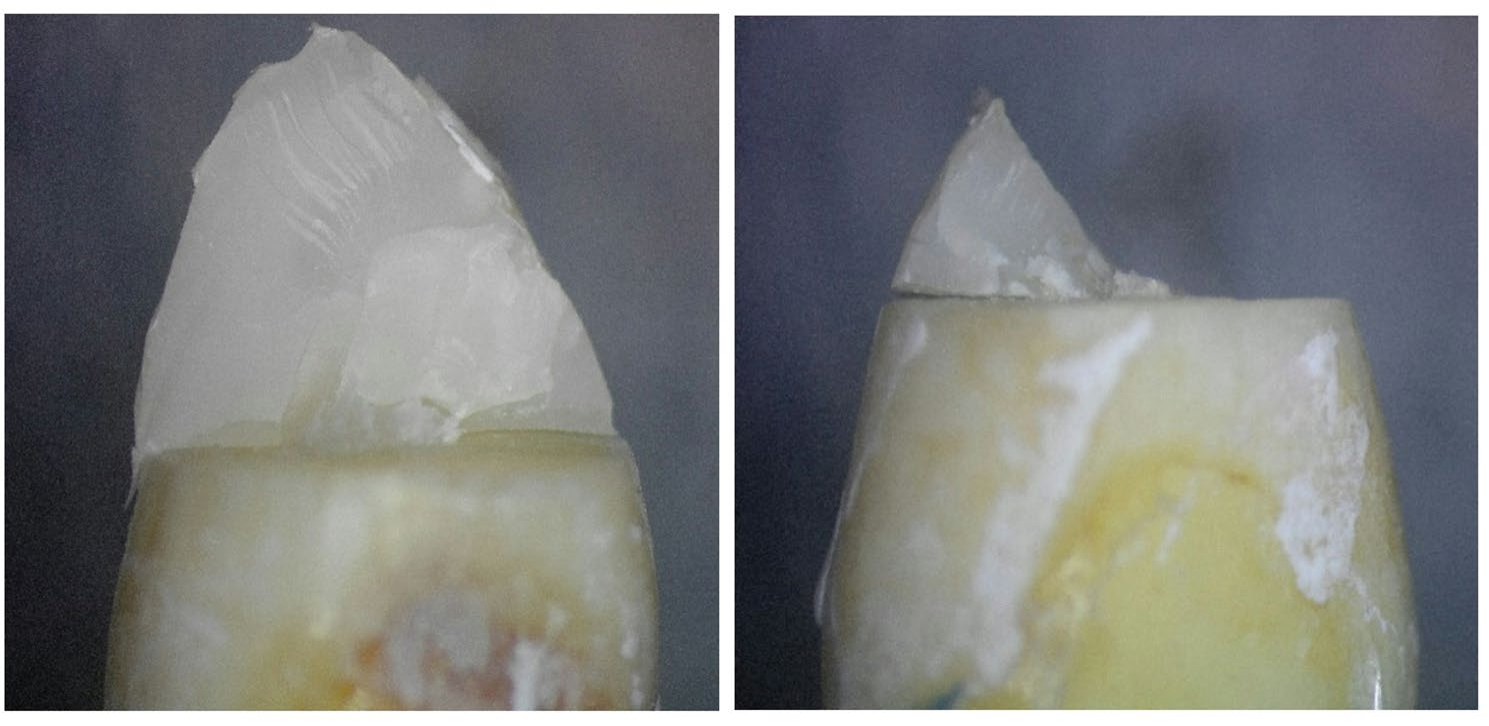
3D Printed group (3D) showing core fractured mostly by macro-crack propagation without involving the residual coronal dentin thus resulting in a favorable failure mode

**Fig. 9:**
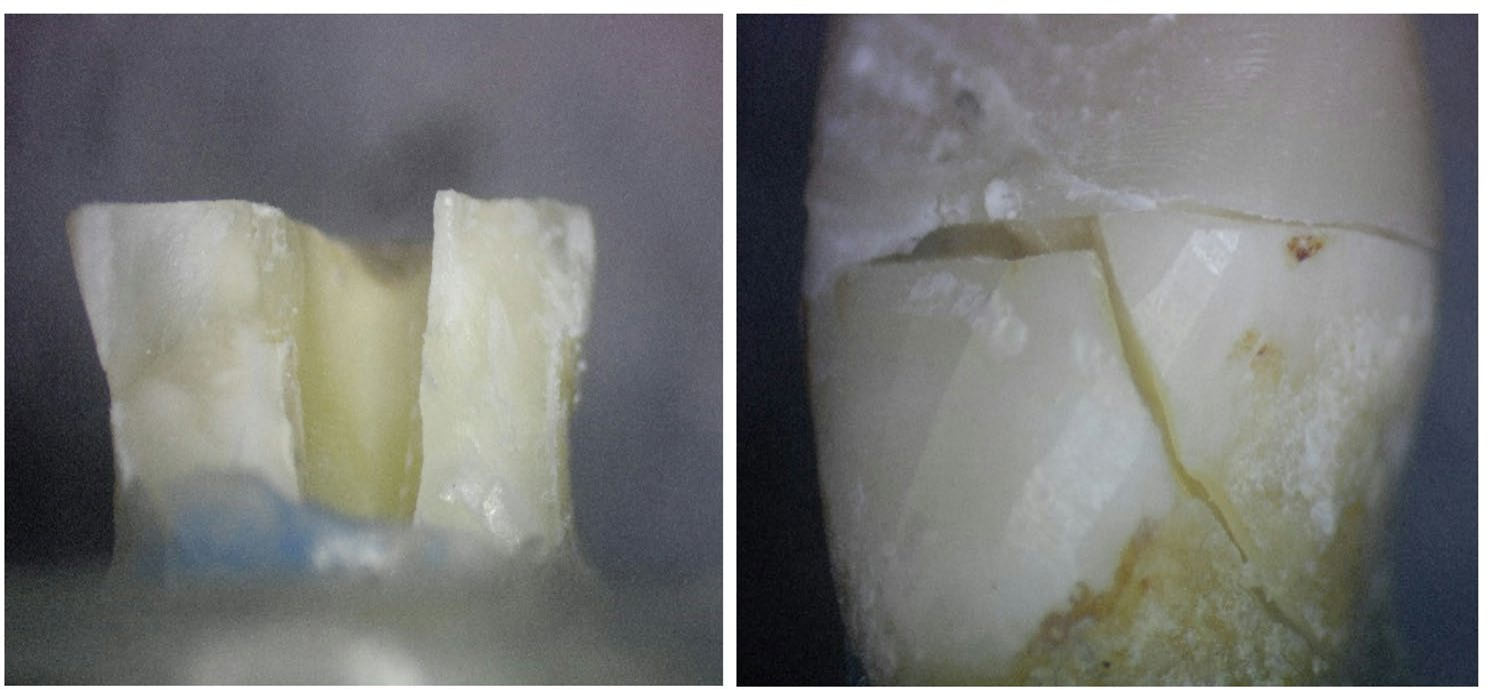
Milled group (MP) showing tooth fracture beneath cemento enamel junction may be due to the flexure of the one-piece post resulting in greater stress on the tooth, leading to non-restorable failure

**Fig. 10:**
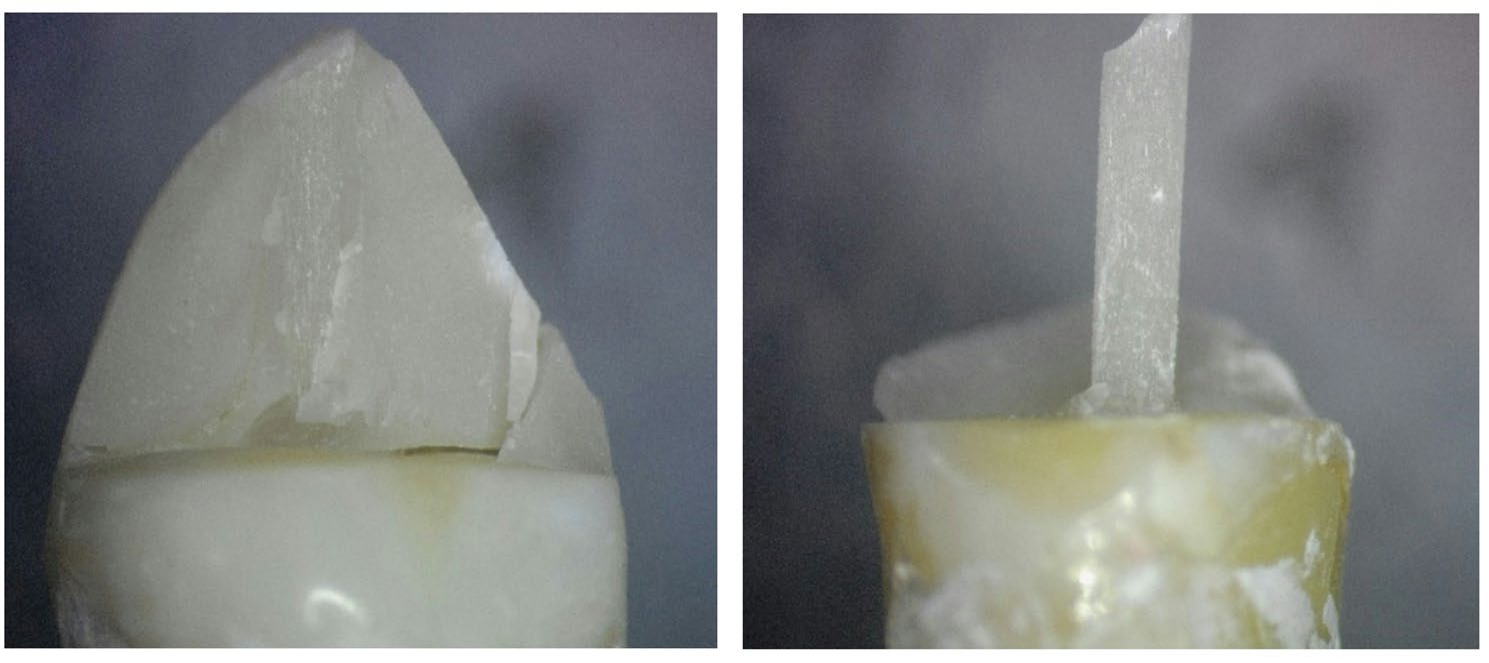
Customized fiber post group (CF) showing separation between post and core and fractured core, improving stress distribution along the root and reducing risk of unrestorable failure

The 3D Printed group showed the highest prevalence of restorable failure mode, followed by the customized fiber post group, then the MP group, which showed the lowest prevalence of restorable failure mode. This was demonstrated in the stereomicroscopic photos (Figs. 8, 9 and 10). However, there was no statistically significant difference between the three groups (P-value = 0.381, effect size = 0.378).

#### Comparison between modes of failure within each group

A one-sample proportion test was used to compare the modes of failure within each group. The significance level was set at *P* ≤ 0.05 (Table 3). There was no statistically significant difference between the prevalence of the two modes of failure within each group (P-value = 0.125, P-value = 1, and P-value = 0.453, respectively.

## Discussion

Restoration of endodontically treated teeth is challenging due to extensive structural loss, and although post–core–crown systems are traditionally used, they may weaken root dentin and increase the risk of perforation [35, 36]. Prefabricated fiberglass posts offer favorable stress distribution but may lead to microleakage due to their high flexibility [37–39]. With advances in digital dentistry, CAD-CAM and 3D printing technologies have enabled efficient fabrication of restorations, with 3D printing offering a cost-effective alternative [40]. VarseoSmile Crown Plus is a new ceramic-filled hybrid resin used for permanent restorations, but its application as a post–core system has not yet been evaluated. Therefore, the present study investigated the fracture resistance of maxillary central incisors that had undergone endodontic treatment and been restored with CAD/CAM-fabricated post and core systems, constructed using subtractive and additive techniques, compared with the standard customized fiber post in terms of fracture resistance and mode of failure.

Natural teeth were utilized to accurately replicate the actual canal space and facilitate proper endodontic treatment. In this study, maxillary central incisors were chosen because they possess ideal root characteristics: long, straight roots with sufficient bulk all around and a circular root canal cross-section, minimizing the influence of varying post geometries on fracture resistance and bonding outcomes [41]. The use of crowns is known to affect the bonding performance of post-and-core systems by providing additional protection against catastrophic failures. Therefore, no extracoronal restorations were included in this study [6].

The null hypothesis of this study was accepted in light of the findings. The data from this study showed that there was no statistically significant difference between the fracture resistance values of the three groups. The customized fiber post (CF) group exhibited the highest mean fracture resistance (321.9 N), followed by the Milled (MP) group (301.6 N), and the 3D printed group, which showed the lowest mean fracture resistance (224.8 N), with no significant difference between the groups.

The customized fiber post (CF) group exhibited the highest fracture resistance, which can be attributed to the modulus of elasticity of glass fiber posts being closely aligned with that of radicular dentin. This similarity facilitates the formation of a monoblock structure, effectively dissipating forces along the post’s long axis and enhancing root resistance to fracture under occlusal loads. Furthermore, fiber-reinforced materials derive their mechanical properties from both the matrix and fibers, as well as the strength of their interfacial bond. A strong interfacial bond ensures efficient load transfer from the matrix to the reinforcing fibers, thereby enhancing the overall mechanical performance of the composite material [42].

This finding is in agreement with a study by Habibzadeh et al. in 2017 [43]. Study has shown that customizing the post improves its adaptation to the root walls while reducing the thickness of the resin cement. This can be accomplished through a fiberglass post relining technique using a microhybrid composite, which precisely replicates the root canal anatomy. Optimal post adaptation enhances frictional retention, thereby improving the overall performance of post-and-core systems.

However, this contradicts another study by Grandini et al. in 2005 [44], study indicates that milling a single-piece post and core reduces the risk of failure, as manual relining with prefabricated posts and composite resin may result in gaps between the post and the resin composite.

Although not statistically significant, the lower fracture resistance of the VarseoSmile 3D-printed group may be attributed to its relatively low filler content (approximately 30–50 wt% inorganic fillers with a 0.7 μm particle size) and its low modulus of elasticity (4.09 GPa). Additionally, the high probability of residual monomers in the 3D-printed restoration may influence bonding [28]. This finding aligns with the results of Grzebieluch et al. in 2021 [40], It has been reported that VarseoSmile, the material with the lowest stiffness, also has the lowest filler content. To maintain a liquid consistency suitable for 3D printing, the filler content is limited, resulting in a relatively low amount of filler in VarseoSmile. However, the material remains stable, with the filler evenly distributed in the resin, preventing sedimentation at the bottom of the vat. Despite this stability, the low flexural modulus of VarseoSmile—significantly lower than that of hard tissues—should be considered a drawback.

Regarding 3D-printed resin technology, the inclusion of fillers increases the viscosity of the resin, potentially leading to printability issues such as clogging, uneven flow, and reduced printing accuracy. Additionally, fillers tend to settle over time, leading to uneven distribution within the resin and resulting in inconsistent mechanical properties in printed objects. Due to the relatively low filler content and the low modulus of elasticity of VarseoSmile (4.09 GPa) compared to hard dental tissues, these factors may explain the lack of significant improvement in fracture resistance outcomes [45–47].

In addition, no significant differences were observed among the three groups tested, likely due to the adhesive resin cement used for post cementation. This finding is in line with the study by Abdel-Aziz et al. in 2023 [48], who found that adhesive resin cement enhanced post retention and optimized fracture patterns, even with shorter post lengths.

The results of the present study are consistent with previous studies; however, other studies reported higher fracture resistance values. This may be due to differences in the materials used, the customization techniques applied, the angulation of the load applied to the long axis of the tooth, or the type of teeth tested [6, 33, 49].

It is important to highlight that the fracture resistance values recorded in this study exceeded the typical occlusal force range in adult anterior teeth, which varies between 190 and 290 N, as reported by Tortopidis et al. in 1998 [50]., forces between the incisors range from 140 to 200 N, while forces between the canine teeth range from 120 to 350 N. teeth [50].

In this study, the mode of failure was classified as favorable, occurring above the CEJ, and unfavorable, which included cracking and fractures apical to the CEJ [33]. The results showed that the 3D printed group (3D) had the highest prevalence of restorable failure mode (85.7%), followed by the customized fiber post group (CF) (71.4%), and then the Milled group (MP), which had the lowest prevalence of restorable failure mode (42.9%). However, no statistically significant difference was found among the three groups (P-value = 0.381, Effect size = 0.378). One possible explanation for this outcome is that the materials used in all three groups have comparable moduli of elasticity, closely matching that of dentin. This similarity enhances load distribution, minimizing the likelihood of catastrophic failures. Additionally, the modulus of elasticity may have played a role in reducing the occurrence of non-repairable fractures. These findings align with the study conducted by Fathey et al. in 2024 [51]. It was concluded that no significant differences were found among the tested groups regarding the restorability of teeth after failure.

Although the CAD/CAM Brilliant Crios group showed the lowest percentage of restorable failure mode (42.9%) and the highest percentage of non-restorable failure mode (57.1%), there was no statistically significant difference between the two modes of failure within this group. This could be attributed to the fact that the one-piece CAD/CAM post and core system reduces the interfaces between the post, the relining composite, and the composite resin core in the coronal area. However, when minimal or no coronal tooth structure remains, post flexure can generate increased stress on the tooth, raising the risk of premature failure [52, 53].

This study has several limitations that should be acknowledged. The results are influenced by the specific materials, protocols, and testing conditions used, which may not fully represent clinical behavior. As an in vitro study, the complex biomechanical and biological conditions of the oral environment could not be completely reproduced. Although thermocycling was performed, dynamic mechanical loading was not applied, limiting simulation of functional fatigue and masticatory stresses. Full-coverage crowns were intentionally excluded to isolate the behavior of the post–core systems; however, this may have influenced force distribution and does not reflect the typical clinical scenario. Additionally, long-term aging effects, interfacial bond durability, and different adhesive strategies were not evaluated, and the sample size may limit the generalizability of the findings. Future studies should incorporate larger sample sizes, cyclic loading protocols, simulated aging, and full-coverage restorations, as well as compare different adhesive and luting protocols. Ultimately, clinical trials are required to confirm the long-term performance of 3D-printed materials such as VarseoSmile Crown Plus for custom post–core applications in CAD/CAM dentistry.

## Conclusion

Within the limitations of this in vitro study, the following conclusions can be drawn:

1. One-piece post and core systems can be successfully milled from indirect composite blocks or printed using 3D printable materials with CAD/CAM technology.
2. All post systems demonstrated a comparable mode of failure, The highest prevalence of restorable failures was observed in both the 3D printed and customized post groups, indicating their potential to preserve tooth structure under simulated loading conditions.

### Clinical significance

Milled composite blocks and 3D-printable materials effectively produced one-piece post-and-core structures using CAD/CAM technology with fracture resistance within the typical masticatory forces for maxillary anterior teeth.

## Data Availability

The datasets used and/or analyzed during the current study are available from the corresponding author on reasonable request.

## Abbreviations

Bis-EMA: Ethoxylated bisphenol A-glycol dimethacrylate
Bis-GMA: Bisphenol A-glycidyl methacrylate
UDMA: Urethane dimethacrylate
TEGDMA: Triethylene glycol dimethacrylate; SiO2, silicon dioxide
CEJ: Pemento-enamel junction
VS: VarseoSmile Crown Plus
